# Virtually Wall-Less Tubular Sponges as Compartmentalized Reaction Containers

**DOI:** 10.34133/2019/4152536

**Published:** 2019-05-30

**Authors:** Shaohua Jiang, Viktoria Gruen, Sabine Rosenfeldt, Anna S. Schenk, Seema Agarwal, Zhi-Kang Xu, Andreas Greiner

**Affiliations:** ^1^College of Materials Science and Engineering, Nanjing Forestry University, Nanjing 210037, China; ^2^Universität Bayreuth, Physical Chemistry, Universitätsstrasse 30, 95447 Bayreuth, Germany; ^3^Universität Bayreuth, Macromolecular Chemistry, Bavarian Polymer Institute, Universitätsstrasse 30, 95440 Bayreuth, Germany; ^4^MOE Key Laboratory of Macromolecular Synthesis and Functionalization, Key Laboratory of Adsorption and Separation Materials & Technologies of Zhejiang Province, Department of Polymer Science and Engineering, Zhejiang University, 310027 Hangzhou, China

## Abstract

Sponges are open cellular materials with numerous interesting features. However, the potential of compartmentalized sponges has not been explored although many new properties and applications could be envisioned. We found that compartmentalized fibrous ultraporous polymer sponges with superhydrophobic surfaces could be designed as virtually wall-less reaction containers. With this, for example, the efficient removal of CO_2_ from water and the controlled mineralization of calcium carbonate are possible. The high porosity (>99%) and superhydrophobicity make these sponges ideal candidates to hold alkanolamine solution for absorbing CO_2_ and exchange gas through the walls of the sponges. The tubular sponge exhibits a much higher evaporation rate than a glass tube with the same diameter due to the much larger contact area between water and air. Therefore, the spongy reaction container also possesses a much faster adsorption rate, smaller equilibration time and higher efficiency for CO_2_ adsorption than the glass tube container. In addition, these tubular sponges are also utilized to precipitate calcium carbonate by ammonium carbonate decomposition, which can control the deposition rates and products by tailoring the porosity and surface chemistry in the future. These new sponges provide an ideal basis for numerous new applications, for example, as breathable pipe lines for gas-liquid exchange, slag slurry carbonization, humidifier, and blood enricher.

## 1. Introduction

Open cellular fibrous materials are omnipresent in nature [1] and offer unique structure property relationships for man-made three-dimensional materials with numerous perspectives for applications. The most prominent examples of fibrous open cellular materials with interconnected pore structures are sponges [2]. Man-made sponges can be prepared following different concepts based on various materials [3, 4]. Ultralight sponges (density > 10 mg/cm^3^, porosity > 99%) made of electrospun fibers are of particular interest. Recently, several groups reported on ultralight sponges based on the self-assembly of short electrospun polymer fibers from freeze-dried dispersions of the fibers [5–7] which provided numerous new property profiles. The conformal coating of ultralight sponges by chemical vapor deposition (CVD) of poly(p-xylylene) (PPX) resulted in superhydrophobic properties with improved mechanical integrity, solvent stability, and excellent insulation properties [8]. Further modification of ultralight sponges based on electrospun fibers could be extended to numerous new functions including drug release [9], tissue engineering [7, 10–14], oil-water separation [5, 6, 15], high temperature shielding [16, 17], electric conductivity [18], thermoresponsivity for water management [19], gelation agent [20], and catalysis for reactivity tuning [21].

**Figure 1:**
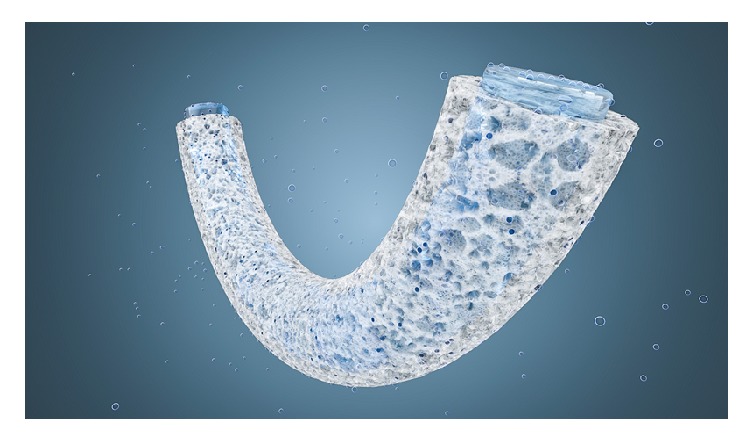
Schematic drawing of a tubular porous sponge. Gas bubbles penetrate the porous wall and can diffuse from the outer atmosphere towards the inner reaction channel.

In this contribution, we explored PPX-coated sponges with tubular shape. As a result, ultralight, low density, and open cellular tubes with highly porous walls with one or two open ends were obtained. These hollow sponges can hold aqueous solution without leakage although the walls are highly porous. The porosity is more than 99.5% which implies the walls are nothing but the network of open holes. The walls of these hollow sponges allow the free exchange of gas between the outside atmosphere and aqueous solution inside the sponge as shown schematically in Figure 1. Containers or vessels with highly permeable walls for gases play an important role in nature, e.g., in lung and blood vessel and in technical systems, e.g., gas exchange membranes. In order to probe the versatility of these novel type of virtually wall-less hollow sponges for gas exchange we investigated qualitatively and quantitatively the sponges as a reaction containers for the efficient removal of gaseous CO_2_ which is a major issue in technical plants [22–26] and for controlled mineralization of calcium carbonate where surface area may play a crucial role [27].

## 2. Results

### 2.1. Preparation and Morphology of Tubular Sponges

**Figure 2:**
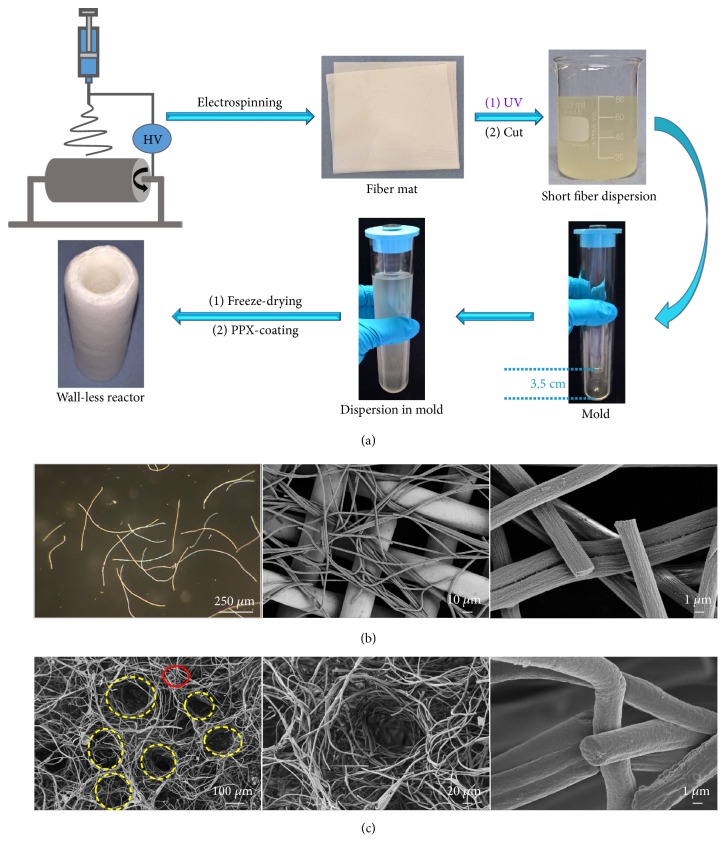
*Concept for the superhydrophobic porous reaction container.* (a) Illustrations of the preparation process leading to hollow sponges from electrospun fibers. (b) Morphology of the short electrospun fibers images by light microscopy and scanning electron microscopy. (c) Scanning electron micrographs showing the morphology of the hierarchical porous structures of the wall of the SG. Dashed circles with yellow and red color indicate the big and small pores, respectively.

We designed tubular sponges as virtually wall-less reaction container following the process shown in Figure 2. The first step was the synthesis of a UV cross-linkable poly(methylacrylate-co-methyl methacrylate-co-4-methacryloyloxybenzophenone) (poly(MA-co-MMA-MABP)) followed by electrospinning of poly(MA-co-MMA-MABP) together with 14 wt% of polyacrylonitrile (PAN) and UV-crosslinking of the obtained fibers. The cross-linked fibers were cut to short fibers in dioxane which resulted a dispersion of short fibers (concentration of fibers = 4.7 mg/mL). The short fibers had an average fiber diameter of 1973 ± 185 nm and a length of 622 ± 279 *μ*m (Figure 2(b)). Then the dispersion was transferred into a glass mold with one glass rod fixed in the center as a template for the tubular opening of the sponge. In order to get the container with one side sealed, a 3.5 cm length between the glass rod and the bottom of the glass tube was reserved. The dispersion in mold was frozen at -25°C, and then the glass rod was removed to form the hole in the center of the sponge. After drying at 0.34 mbar for 48 h, the as-prepared sponge (density: 5.69 mg/cm^3^) with hollow column in the center and one end sealed was coated with a thin layer of PPX by CVD. After coating, the fiber diameter increased to 2664 ± 289 nm, implying an approx. 345 nm thickness of PPX coating around the fibers (Figure 2(c)). The PPX-coated hollow sponges (SG) showed an increased density of 9.26 mg/cm^3^. The wall of the SG displayed hierarchical pore structure with big pores between the assembled aggregation of fibers (circles with yellow dashed line) and small pores in between the fibers (circle with red solid line) (Figure 2(c)). In spite of the low density and the highly porous character SG is mechanically very stable as demonstrated by manual squeezing ([Supplementary-material supplementary-material-1]).

### 2.2. Superhydrophobicity of SG

**Figure 3:**
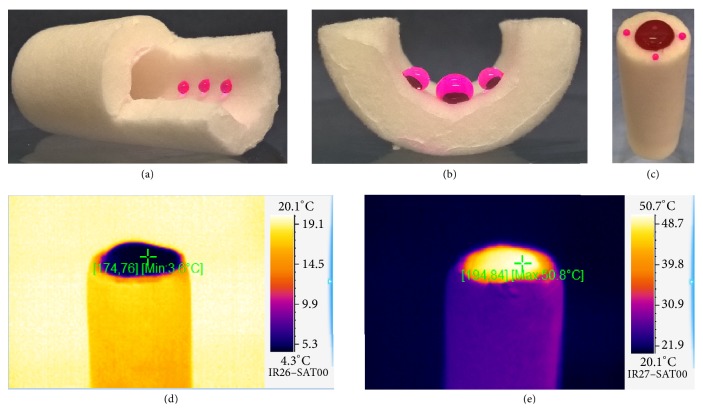
*Proof for superhydrophobic SG sponge walls. *(a) Photograph of small water droplets dyed by phenolphthalein inside the SG (opened by cutting). (b) Photograph of a cross-sectional view on the inside of a SG with big water droplets dyed by phenolphthalein. (c) Photograph of full water column (dyed with phenolphthalein) filling the center of the SG. (d) Thermal image of SG (one end closed) filled completely with cold water (dyed with phenolphthalein). (e) Thermal image of a SG (one end closed) filled completely with warm water (dyed with phenolphthalein).

The superhydrophobic nature of the walls of the SG and their penetrability for water under atmospheric pressure was demonstrated by the placements of dyed water droplets on the inner side of the SG (Figures 3(a) and 3(b)). Even after complete filling of the center of the SG with dyed water no penetration of water through the walls was observed (Figure 3(c)). Cold water as well as hot water clearly does not penetrate the wall of the SG under atmospheric pressure as shown by thermal images (Figures 3(d) and 3(e)).

### 2.3. Gas-Liquid Exchange Capability of SG

**Figure 4:**
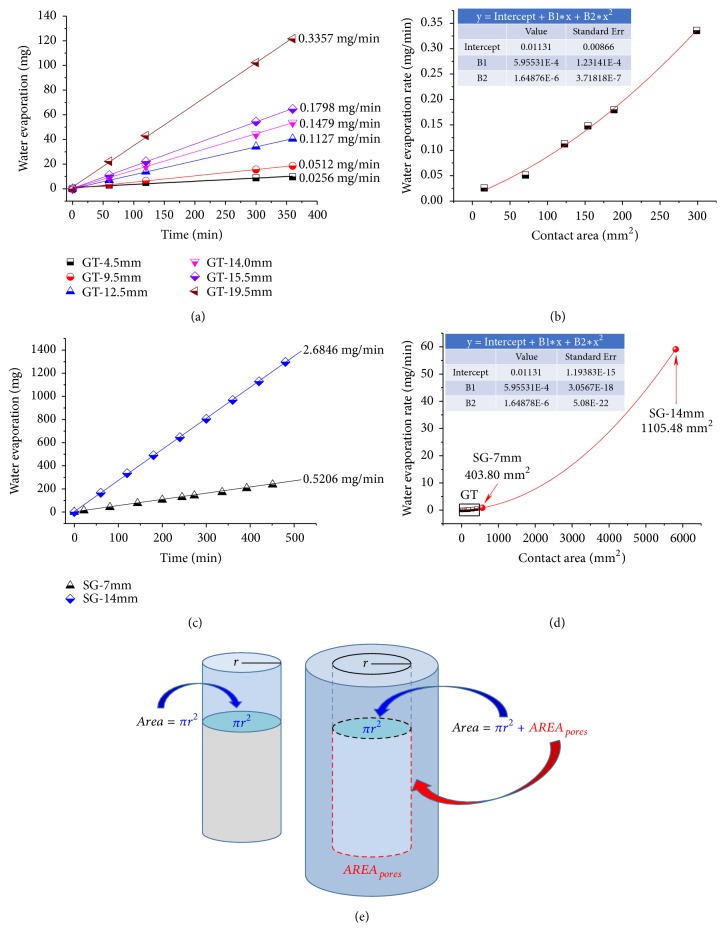
*Liquid water evaporation at 20*°*C from inside GT and SG.* (a) Time dependent water evaporation from glass tubes (GT) with different inner diameters. (b) Polynomial fitting on the water evaporation rate in GT and the contact area of water to air. (c) Time dependent water evaporation from SG. (d) Corresponding contact area of water to air in SG according to the polynomial fitting. (e) Illustration of the contact area of water to air in glass tube and hollow sponge.

The hierarchical porous structures of the SG wall provide exceptional path for gas exchange between the inside of the SG and the outer atmosphere which was proven experimentally by evaporation of liquid water from the inside of the SG with different inner diameter (7.0 and 14.0 mm). For comparison, standard glass tubes (GT) with 1 open end, which naturally have solid walls, with different inner diameters (4.5, 9.5, 12.5, 14.0, 15.5, and 19.5 mm) were used for the water evaporation experiment. As expected, the water evaporation was highly dependent on the diameter of the GT (Figure 4(a)). As the diameter of the GT increased from 4.5 to 19.5 mm, the water evaporation became faster and the corresponding evaporation rate increased from 0.0256 to 0.3357 mg/min. Due to the constant pressure (1 atm) and temperature (20°C), the difference of the evaporation rate could be attributed to the increased contact area of water to air. Figure 4(b) shows the relationship between the water evaporation rate and the contact area. The relationship could be described by a polynomial fitting with mono basic quadratic equation very well. In contrast, the SG exhibited significantly faster water evaporation than GT. The relationship between the amount of water evaporation and time was linear as well for SG (Figures 4(c) and 4(d)). The SG with inner diameter of 7 and 14 mm presented water evaporation rate of 0.5206 and 2.6846 mg/min, which were 3.5 and 18 times of that from GT with inner diameter of 14.0 mm and even 55% and 700% higher than that from GT with inner diameter of 19.5 mm. This by several orders of magnitude higher water evaporation rate from SG could be due to the larger contact area of water to air (Figure 4(e)). According to the polynomial fitting equation from Figure 4(b), the corresponding contact areas of water to air for the SG with inner diameter of 7 and 14 mm were 403.80 and 1105.48 mm^2^, respectively, which are 162% and 618% higher than those of the GT with inner diameter of 14.0 mm.

**Figure 5:**
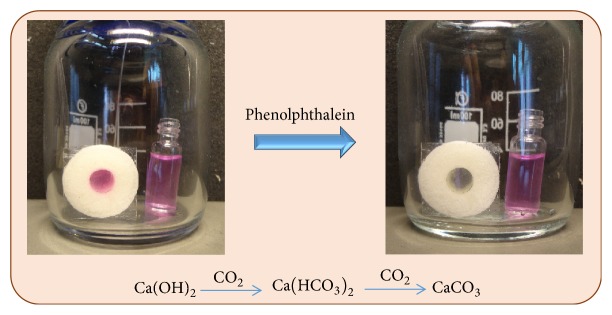
*Proof of gas-liquid exchange.* Neutralization reaction between CO_2_ and Ca(OH)_2_ monitored with phenolphthalein as a pH-sensitive indicator within a wall-less reaction container as compared to a glass vial.

In order to verify the excellent gas-liquid exchange capability of SG further in combination with a chemical reaction we performed a qualitative but very instructive experiment on CO_2_ diffusion through the walls of the SG reaction container ([Supplementary-material supplementary-material-1]). As shown in Figure 5, 1.20 mL of saturated Ca(OH)_2_/water solution with light red color dyed with phenolphthalein was filled in the SG and a standard glass vial for comparison. Both sides of the SG were sealed with a cover glass while the vial was open on the top. After the CO_2_ was purged inside for approx. 2 min, the color of the solution in the SG became colorless indicating the alkaline solution became neutral or acidic solution due to the neutralization of Ca(OH)_2_ with CO_2_. In comparison, the solution in vial remained red indicating the solution was still alkaline. The difference on the reaction rate in the sponge reactor and in the vial could be attributed to the reaction contact area that the solution in the sponge possessed much higher contact area from the porous wall of the sponge while the solution in the vial only had the contact area on the top. This statement has to be proven quantitatively which was achieved by implementing the following set of experiments.

**Figure 6:**
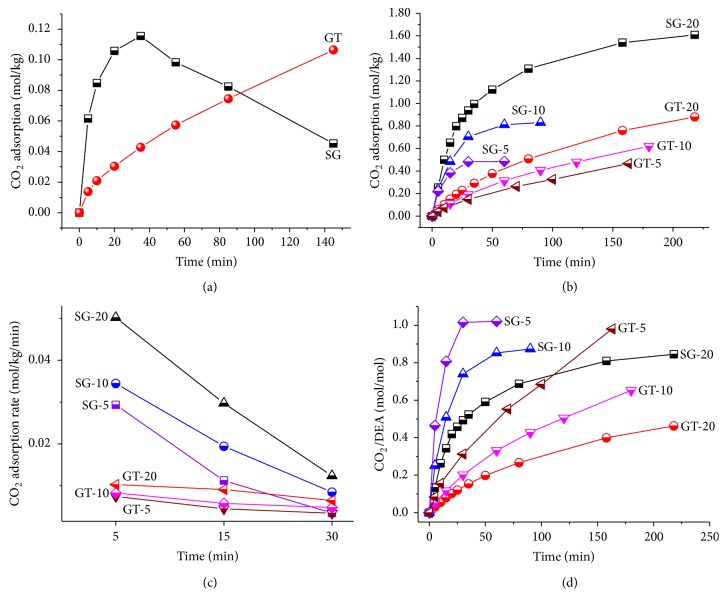
*CO*_*2*_ *adsorption with SG and GT as containers at pressure of 5 bar.* (a) CO_2_ adsorption by water with SG and GT as container. (b) CO_2_ adsorption by DEA solutions with different concentrations of 5, 10, and 20 wt% with SG and GT as container. (c) CO_2_ adsorption rate at 5, 15, and 30 min. (d) CO_2_ solubility in DEA solutions with SG and GT as container.

Verification of the above initial finding quantitatively with technically highly relevant removal of CO_2_ by diethanolamine (DEA) [28–31] in SG and GT both with inner diameter of 14.0 mm was performed at 5 bar and 20°C in an autoclave. The amount of CO_2_ adsorption was determined by measuring the difference of the weight before and after the adsorption by an analytical balance with readability of 0.0001 g. Before the reaction, a blank experiment with water in SG and GT was carried out to evaluate the adsorption of CO_2_ (Figure 6(a)). SG as container showed a fast initial CO_2_ adsorption rate than the vial due to the quick gas transfer through the pores of SG. At the first 5 min, SG container exhibited CO_2_ adsorption rate of 0.00756 mol/kg/min, which was nearly 4.0 times of that with GT as container (0.00192 mol/kg/min). Interestingly, during the observation time of 145 min, the CO_2_ adsorption in SG container firstly increased. The highest CO_2_ adsorption of 0.115 mol/kg was reached at 35 min, at which a saturation of CO_2_ adsorption by water in SG was obtained. After that, the adsorption of CO_2_ dropped down, which could be attributed to the fast release of CO_2_ when the SG container was taken out for weight measurement.

Further experiment on CO_2_ adsorption by DEA with concentrations of 5, 10, and 20 wt% in SG and GT was carried out at 5 bar and 20°C in an autoclave. The time dependent CO_2_ adsorption and adsorption rate were illustrated in Figures 6(b) and 6(c) and Table 1. Due to the much higher adsorption of CO_2_ by DEA than water, herein the CO_2_ adsorption by the water in DEA solution was negligible. As expected, with the same DEA concentration, SG as reaction container exhibited much faster adsorption rate and equilibrium than GT. Taking 5 wt% DEA solution, for example, SG-5 only took 30 min while GT-5 needed more than 150 min to achieve the reaction equilibrium and the reaction rate in SG was 4 times of that in GT. These suggested the much higher efficiency of CO_2_ adsorption by SG than GT. When we normalized the CO_2_ adsorption efficiency by the amount of DEA, interestingly, the DEA solution with smaller concentration showed faster CO_2_ adsorption rate and in general CO_2_ adsorption rate with SG as container was faster than that with GT as container (Figure 6(d)).

### 2.4. Controlled Mineralization

**Figure 7:**
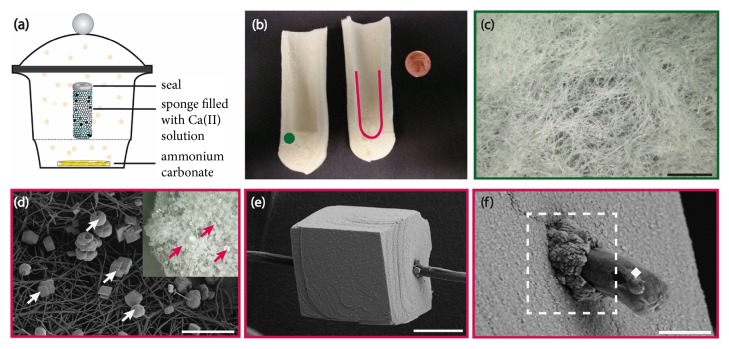
*Calcium carbonate precipitation within the tubular sponge reactor.* (a) Schematic illustration of the gas-diffusion setup used for CaCO_3_ precipitation within hollow polymer tubes. (b) Photograph showing the longitudinal cross section of a sponge reactor after mineral deposition. The green dot marks the sponge wall, while the red line indicates the inner wall surface exposed to the reactant solution. (c) Light microscopy image of the polymer fibers within the tube wall (green dot in (b), scale bar = 200 *μ*m). (d) Scanning electron micrograph showing calcium carbonate particles (indicated by white arrows) grown on the inner wall of the reaction channel (red delineation in (b), scale bar = 100 *μ*m). Inset: overview image obtained by light microscopy demonstrating the full coverage of the channel surface with mineral particles (red arrows). (e) Rhombohedral calcium carbonate crystal grown around a polymer fiber (scale bar = 10 *μ*m). (f) Scanning electron micrograph showing the position, where a polymer fiber (white diamond) protrudes from a calcium carbonate crystal. Small nanoscale particles mediate the contact between polymer and mineral within the interfacial region (dashed box, scale bar = 2 *μ*m).

To illustrate the use of our tubular sponges as highly versatile reaction containers in interface-mediated gas-diffusion reactions, we deposited calcium carbonate within the inner column of SG* via* the widely used ammonium carbonate diffusion technique [27]. For that purpose, SG filled with a reactant solution containing CaCl_2_ and poly(acrylic acid) was exposed to a vapor phase composed of NH_3_ and CO_2_ formed by gradual decomposition of (NH_4_)_2_CO_3_ at room temperature in a closed reaction chamber (Figure 7(a)). After completion of the reaction, the inner surface of the SG (Figure 7(b), red delineation) as well as its fibrous wall of (Figure 7(b), green dot) was examined. The solid precipitation appeared to be completely inhibited within the porous network of the container wall (Figure 7(c)). Energy-dispersive X-ray analysis (EDX) confirmed the absence of calcium, while CaCO_3_ crystals were observed in large quantities in association with the polymer fibers at the inner surface of the reactor (Figures 7(d)–7(f)). The mineral particles deposited within the channel were mainly spherical or rhombohedral in shape (Figure 7(d)) and comprised a mixture of calcite and vaterite (C:V ≈ 88:12, [Supplementary-material supplementary-material-1]). These ~20 *μ*m-sized crystals appeared to be threaded onto the sponge fibers (Figure 7(e)), where the interfacial contact was mediated by very small mineral particles (~200 nm) tightly associated with the polymer surface (Figure 7(f)).

Poly(acrylic acid) as a structure-directing additive has previously been shown to promote mineral precipitation according to a polymer-induced liquid precursor (PILP) mechanism [32, 33], where the polyelectrolyte stabilizes a highly hydrated amorphous precursor phase of calcium carbonate, which can wet surfaces [33] and infiltrate submicrometer pores by capillary action [34] before eventually crystallizing upon dehydration [35].

In our experiment carried out in virtually wall-less sponge reactors, however, the liquid-like mineral precursor, which is expected to form as an intermediate under the chosen conditions, was effectively prohibited from penetrating deep into the porous network due to the superhydrophobicity of the polymer fibers. Hence, in support of our hypothesis, mineral formation was restricted to the inner reaction channel of the hollow sponge container and the fibers themselves were nonwettable by the liquid-like amorphous mineral precursor. EDX mapping revealed that calcium ions were accumulated exclusively within the larger crystals and there was no signal attributed to the Ca K*α*-line detected in the adjacent areas of the fiber ([Supplementary-material supplementary-material-1]). Therefore, nucleation of crystalline calcium carbonate occurred only locally in distinct spots on the fiber surface, which presumably coincide with small-scale defects in the PPX coating.

## 3. Discussion

In this work, we demonstrated a novel fibrous virtually wall-less hollow sponge based on electrospun fibers with high porosity and superhydrophobicity. The sponge can hold alkanolamine solution and exchange gas through the walls of the sponges. Compared to glass tubes with the same inner diameter of 14 mm, the hollow sponges have much faster water evaporation rates, which were 18 times of that from the glass tubes. The sponge as reaction container presents much faster adsorption rate to CO_2_, which are up to 5 times of the reaction container of glass tubes when using the concentration of the diethanolamine in the range of 5-20 wt%. It could be envisioned that highly porous superhydrophobic sponges with numerous tubular channels could be utilized for highly efficient technical air purification of CO_2_ or other acidic gases by diethanolamine or related reagents. The excellent gas permeability of the virtually wall-less sponge has additionally been utilized in the precipitation of calcium carbonate* via* ammonium carbonate decomposition. As the kinetics of this reaction are strongly governed by the transport of the gaseous reactants across the air/solution interface and thus by the diffusion profile and local supersaturation, the new hollow sponge provides the unique possibility of controlling deposition rates and products by tailoring the porosity and surface chemistry of the fibrous polymer network. Intriguingly, even gradients with respect to gas permeability could be introduced into this highly flexible system by adjusting the processing parameters of the container. These sponges could also find applications in many other technological fields, for example, as humidifier and enrichers for blood.

## 4. Materials and Methods

### 4.1. Materials

Polyacrylonitrile (M_w_ = 150 000, Polyscience Inc.), [2.2] paracyclophane (parylene N, Speciality Coating Systems), dimethyl sulfoxide (DMSO, Fisher Chemical, 99.99%), dimethylformamide (DMF, Fisher Chemical, 99.99%), dioxane (technical grade), and acetone (technical grade), diethanolamine (DEA, 99.0%, Aldrich) were used as received. The monomers, methyl acrylate (MA, Aldrich, 99%) and methyl methacrylate (MMA, Aldrich, 99%) were purified by neutral aluminum oxide column before use. The initiator, 2,2'-azobis (isobutyronitrile) (AIBN, Fluka, 98%), was recrystallized from methanol (Aldrich, 99.8%). The cross-linker, 4-methacryloyloxybenzophenone (MABP), and the polymer, poly(MA-co-MMA-MABP) (M_n_ = 2.43 × 10^5^), were prepared according to our previous report [7].

### 4.2. Preparation of SG

The solution (22 wt%) for electrospinning was prepared by blending 25.28 g of poly(MA-co-MMA-co-MABP), 3.50 g of PAN, 46.50 g of DMSO, 35.50 g of DMF, and 20.50 g of acetone. The electrospinning parameters were high voltage (HV) of 20 kV, flow rate of 1.8 mL/h, collecting distance of 30 cm, and humidity of 40-60%. A rotating drum with diameter of 20 cm and rotation speed of 20 rpm was used to collect the electrospun fiber mats.

The obtained mats were first dried at room temperature for 14 h and then cross-linked by UV light (UV lamp 250GS) with an irradiation distance of 15 cm for 5 h for each side. Afterwards, the short fiber dispersion with concentration of 4.60 mg/mL was prepared by cutting the cross-linked fiber mat in dioxane with a razor blade at a rotation of 5000 rpm for 45 s. 80 mL of the dispersion was filled in the glass mold with inner diameter of 3.0 cm and one glass rod with a diameter of 1.4 cm placed in the middle. The dispersion in the mold was frozen at -25°C and dried by a freeze-drying machine (Christ Beta 2-16) at 0.34 mbar for 48 h to form the as-prepared sponge with hollow column in the middle.

4 pieces of the above sponges were put in the chemical vapor deposition chamber of coating machine. 2.0 g of [2.2] paracyclophane was sublimated at 150°C and become radical monomer gas by pyrolysis at 650°C. The monomer gas was cooled at 20°C under 35 mtorr and formed PPX coating on the fibers surface of the sponges with an average thickness of 390 nm.

### 4.3. Water Evaporation from SG

Different amount of water (1.00, 2.00, 5.00, 10.00, 10.00, and 20.00 g) was stored in GT with inner diameter of 4.5, 9.5, 12.5, 14.0, 15.5, and 19.5 mm while 1.50 and 10.00 g of water were stored in SG with inner diameter of 7.0 and 14.0 mm, respectively. All the above samples were put in a room with constant temperature of 20°C at approx. 1 atm. The weight changes from the water evaporation at different time were determined by an analytical balance with readability of 0.01 mg.

### 4.4. Chemical Reactions in SG

The initial attempt for the CO_2_ involved reaction was performed with Ca(OH)_2_. 1.20 mL of saturated light red Ca(OH)_2_/water/phenolphthalein was filled in one SG container and vial. The SG (inner diameter of 7.0 mm; height of 1.5 cm) were sealed both sides with cover slide while the vial was not sealed by the cap. The SG and vial with Ca(OH)_2_ solution were put in bottle with two needles to provide inlet and outlet of CO_2_.

5, 10, and 20 wt% DEA/water solutions were prepared for the quantitate adsorption for CO_2_ in SG and GT. The SG and GT had the same inner diameter of 14.0 mm. The SG and GT containing 10.0 g of the DEA solutions were sealed in an autoclave in which 5 bar of CO_2_ was applied. After different time, the increased weight of the solutions by the adsorption of CO_2_ was measured by an analytical balance with readability of 0.0001 g at different time interval. With the increased weight from the adsorption of CO_2_, the corresponding molar values of CO_2_ could be obtained.

Calcium carbonate was deposited within a wall-less reaction container by filling the hollow sponge with 10 mL of a 20 mM solution of CaCl_2_ · 2H_2_O (Sigma-Aldrich). Poly(acrylic acid) (10 *μ*g/mL, M_W_ ~100 000 g/mol, 35 wt% in H_2_O, Sigma-Aldrich) was added as a structure-directing additive to promote crystallization* via* a polymer-induced liquid precursor [32, 33]. Precipitation of the mineral phase was induced by exposing the reactant solution to ammonium carbonate vapor within a closed desiccator (Figure 7(a)). The open end of the tubular sponge was sealed such that diffusion of the gaseous reagents into the Ca(II)-solution was restricted to the porous polymer network. After 2 days of reaction time the sponge was removed from the reaction chamber and the inner channel was washed with deionized water and ethanol and subsequently left to dry under ambient conditions. The mineral precipitates formed at the inner wall of the hollow sponge were characterized by optical microscopy (VHX-950F, Keyence) and scanning electron microscopy (LEO 1530 Gemini, Zeiss) in combination with energy-dispersive X-ray spectroscopy (Thermo Fisher Scientific NS7). Powder X-ray diffractograms were recorded in Bragg Brentano geometry as coupled *θ*-2*θ* scans spanning an angular range of 20° < 2*θ* < 85°. Measurements were performed with an Empyrean system (PANalytical, Almelo, Netherlands) equipped with a sealed X-ray tube (Cu-K*α*, Ni-filtered) and a PIXEL solid state detector.

## Figures and Tables

**Table 1:** CO_2_ adsorption rate (mol/kg/min) with SG and GT as containers at different time.

	SG-5	GT-5	SG-10	GT-10	SG-20	GT-20
5 min	0.02933	0.00745	0.03447	0.00827	0.05019	0.01028
15 min	0.01125	0.00449	0.01944	0.00578	0.02972	0.00909
30 min	0.00347	0.00342	0.00848	0.00478	0.01236	0.00649

## Supplementary Materials

Supplementary Materials
* Movie S1*: mechanically stable sponge to resist manual squeezing.* Movie S2*: comparative experiment for the adsorption of CO_2_ using sponge and glass tube as reaction container.* Figure S1: Powder X-ray diffraction analysis of SG.* (a) Diffractograms of the polymer sponges were obtained before (red squares, SG pure) and after (blue circles, SG loaded) mineral deposition. The reaction container in its original state showed only 2 broad halos as they are characteristic of polymeric compounds. In contrast, the sponge piece which had been cut out from the mineralized channel surface after exposure to ammonium carbonate vapor exhibited distinct Bragg peaks indicating the presence of crystalline particles. The PXRD pattern of the deposited calcium carbonate mineral (black line, background corrected) was obtained after subtraction of a normalized SG pure background profile from the curve of the loaded container. (b) After background subtraction the diffraction profile of the mineralized sponge (inner channel surface, black triangles) could be assigned to a mixture of calcite (thick red line) and vaterite (thin blue line) using the software X'Pert High Score Plus 3.0 (PANalytical). The intensities of the (104)-peak of calcite and the (101)-peak of vaterite were used to roughly estimate the ratio of calcite to vaterite. For that purpose, the (104) Bragg peak of calcite was normalized to I(2*θ*) = 100 counts and the resulting pattern was compared with reference data obtained from rhombohedral calcite (ICDD-PDF reference code 01-085-0849) and hexagonal vaterite (ICDD-PDF reference code 00-024-0030) yielding a ratio of ca. 88% calcite and 12 % vaterite.* Figure S2: EDX analysis of a representative area on the inner surface of the sponge reaction container.* (a) Scanning electron micrograph of a calcium carbonate crystal (red asterisk) deposited around polymer fibers within the inner lining of the container wall (white arrows). The contact area between the micron-scale particle and the organic fiber is lined with smaller crystallites (blue circle). (b) Corresponding EDX-map showing the distribution of calcium in the same area. The intensity of the signal attributable to the Ca K*α*-line was mapped in steps of 0.19 *μ*m at an acceleration voltage of 10 kV.
