# Supplementary material for: Virtually Wall-Less Tubular Sponges as Compartmentalized Reaction Containers
Source: Research (Wash D C). 2019 May 30;2019:4152536. doi: 10.34133/2019/4152536 (PMC6750054; doi:10.34133/2019/4152536)
Supplement: Supplementary Materials — Movie S1: mechanically stable sponge to resist manual squeezing. Movie S2: comparative experiment for the adsorption of CO2 using sponge and glass tube as reaction container. Figure S1: Powder X-ray diffraction analysis of SG. (a) Diffractograms of the polymer sponges were obtained before (red squares, SG pure) and after (blue circles, SG loaded) mineral deposition. The reaction container in its original state showed only 2 broad halos as they are characteristic of polymeric compounds. In contrast, the sponge piece which had been cut out from the mineralized channel surface after exposure to ammonium carbonate vapor exhibited distinct Bragg peaks indicating the presence of crystalline particles. The PXRD pattern of the deposited calcium carbonate mineral (black line, background corrected) was obtained after subtraction of a normalized SG pure background profile from the curve of the loaded container. (b) After background subtraction the diffraction profile of the mineralized sponge (inner channel surface, black triangles) could be assigned to a mixture of calcite (thick red line) and vaterite (thin blue line) using the software X'Pert High Score Plus 3.0 (PANalytical). The intensities of the (104)-peak of calcite and the (101)-peak of vaterite were used to roughly estimate the ratio of calcite to vaterite. For that purpose, the (104) Bragg peak of calcite was normalized to I(2θ) = 100 counts and the resulting pattern was compared with reference data obtained from rhombohedral calcite (ICDD-PDF reference code 01-085-0849) and hexagonal vaterite (ICDD-PDF reference code 00-024-0030) yielding a ratio of ca. 88% calcite and 12 % vaterite. Figure S2: EDX analysis of a representative area on the inner surface of the sponge reaction container. (a) Scanning electron micrograph of a calcium carbonate crystal (red asterisk) deposited around polymer fibers within the inner lining of the container wall (white arrows). The contact area between the micron-scale particle and the o [file 4152536.f1.zip › Supplementary figures.docx]

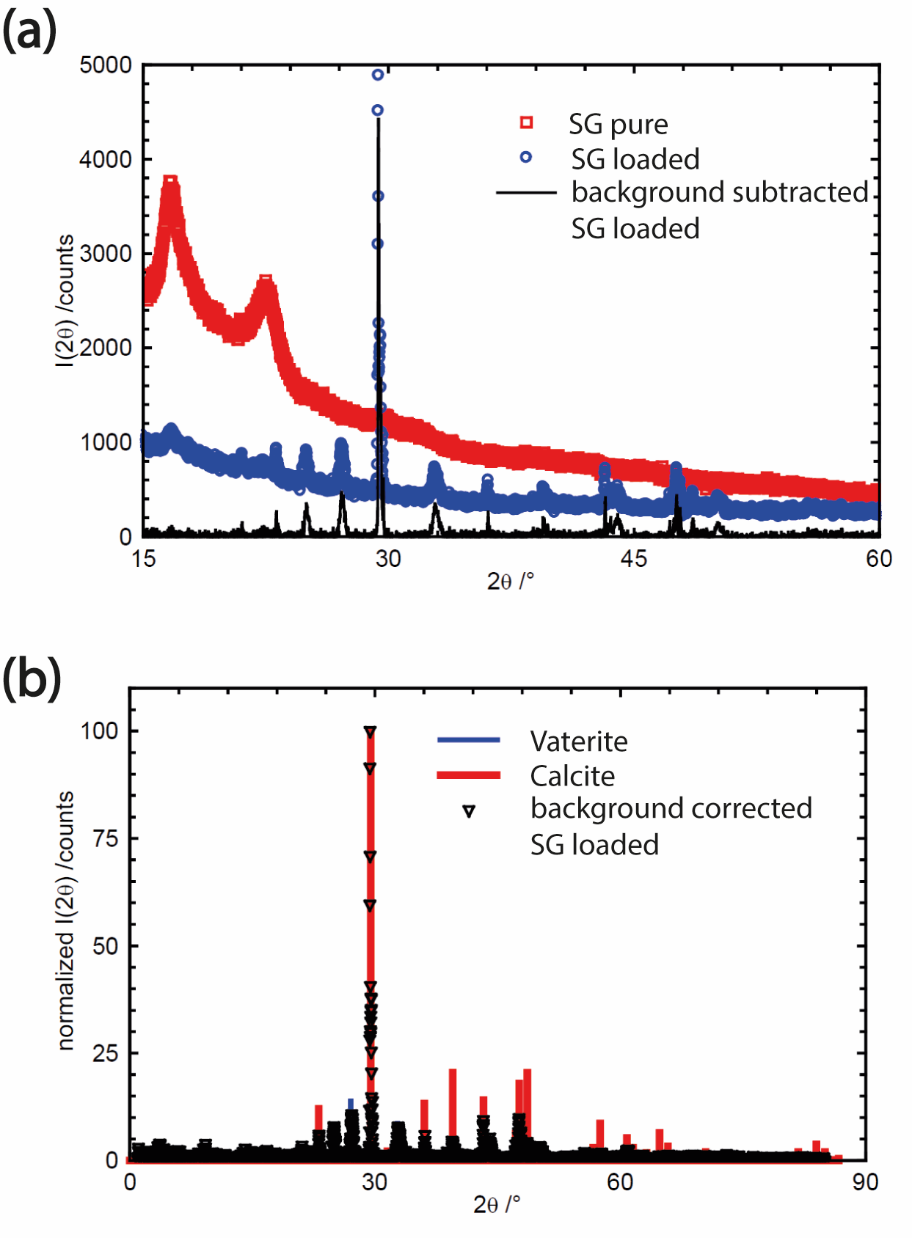


**Figure S1. Powder X-ray diffraction analysis of SG.** (a) Diffractograms of the polymer sponges were obtained before (red squares, SG pure) and after (blue circles, SG loaded) mineral deposition. The reaction container in its original state showed only 2 broad halos as they are characteristic of polymeric compounds. In contrast, the sponge piece which had been cut out from the mineralized channel surface after exposure to ammonium carbonate vapor, exhibited distinct Bragg peaks indicating the presence of crystalline particles. The PXRD pattern of the deposited calcium carbonate mineral (black line, background corrected) was obtained after subtraction of a normalized SG pure background profile from the curve of the loaded container. (b) After background subtraction the diffraction profile of the mineralized sponge (inner channel surface, black triangles) could be assigned to a mixture of calcite (thick red line) and vaterite (thin blue line) using the software X’Pert High Score Plus 3.0 (PANalytical). The intensities of the (104)-peak of calcite and the (101) -peak of vaterite were used to roughly estimate the ratio of calcite to vaterite. For that purpose, the (104) Bragg peak of calcite was normalized to I(2Ɵ) = 100 counts and the resulting pattern was compared with reference data obtained from rhombohedral calcite (ICDD-PDF reference code 01-085-0849) and hexagonal vaterite (ICDD-PDF reference code 00-024-0030) yielding a ratio of ca. 88% calcite and 12 % vaterite.


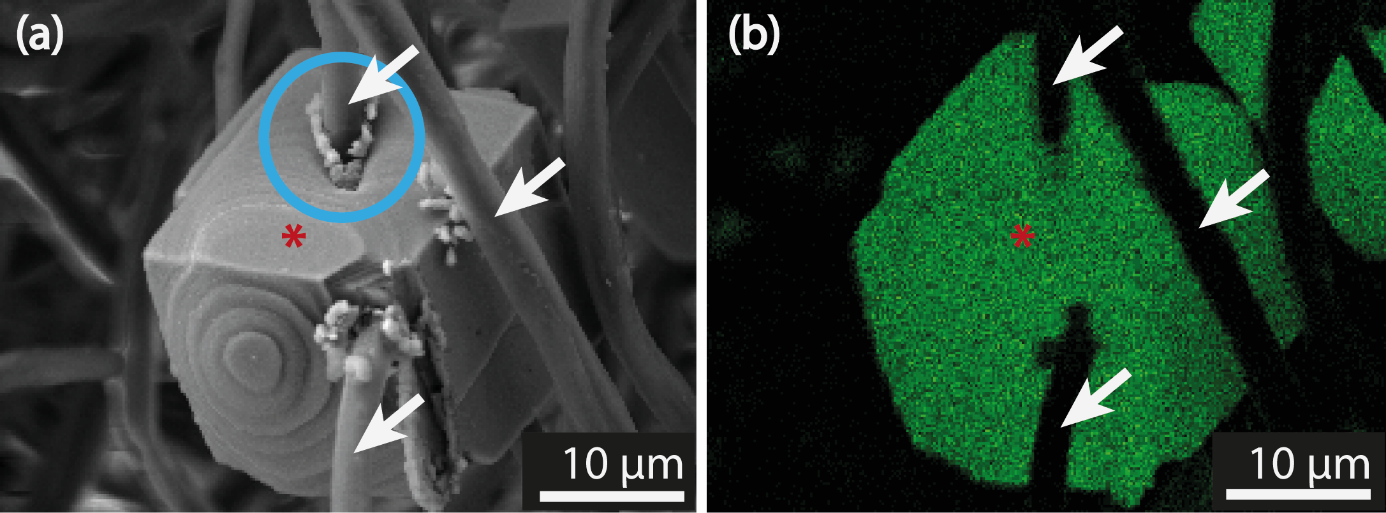


Figure S2. EDX analysis of a representative area on the inner surface of the sponge reaction container. (a) Scanning electron micrograph of a calcium carbonate crystal (red asterisk) deposited around polymer fibers within the inner lining of the container wall (white arrows). The contact area between the micron-scale particle and the organic fiber is lined with smaller crystallites (blue circle). (b) Corresponding EDX-map showing the distribution of calcium in the same area. The intensity of the signal attributable to the Ca Kα-line was mapped in steps of 0.19 µm at an acceleration voltage of 10 kV.
